# IoT and ML-driven framework for managing infectious disease risks in communal spaces: a post-COVID perspective

**DOI:** 10.3389/fpubh.2025.1552515

**Published:** 2025-05-14

**Authors:** Dhruv Parikh, Avaneesh Karthikeyan, V. Ravi, Merin Shibu, Riya Singh, Reka S. Sofana

**Affiliations:** ^1^School of Electronics Engineering, Vellore Institute of Technology, Chennai, India; ^2^Centre for Neuroinformatics, School of Electronics Engineering, Vellore Institute of Technology, Chennai, India

**Keywords:** coronavirus disease, face mask detection, face shield detection, Tkinter GUI, aarogya setu database, internet of things, machine learning

## Abstract

COVID-19 has not only changed the way people live but has also altered the way all organizations operate. The most effective precautionary measure against the spread of the virus that caused the COVID-19 pandemic SARS-CoV-2, is to use face coverings in public settings. In this study, we present a potential application of the Internet of Things (IoT) and machine learning to prevent the spread of COVID-19. The proposed smart gateway entrance system consists of various subsystems: face mask recognition, face shield detection, face mask detection with face shields, sanitization systems, temperature monitoring systems, and vaccine verification. These systems help us to efficiently monitor, authenticate, track health parameters, and process data in real-time. The face mask and face shield detection subsystems leverage a hybrid model that combines the capabilities of MobileNetV2 and VGG19, enabling more robust and accurate detection by leveraging MobileNetV2′s efficiency and VGG19′s depth in feature extraction, which has an overall accuracy of 97% and notably the face shield detection component obtains an efficiency of 99%. Proposed framework includes QR code-based vaccination certificate authentication using a secure real-time database model, inspired by health platforms such as CoWIN, to ensure reliable and timely verification at points of entry and the real-time database management system developed using Haar Cascade trainer GUI helps to integrate all the data in real-time and provides access to the entry. The IoT model sanitizes individuals and tracks health parameters using an MLX90614 infrared sensor with an accuracy of ±0.5°C. As the system updates the real-time database, it helps maintain a record of the employee's health conditions and checks whether the employee follows all safety screening protocols every day. Therefore, the proposed system has immense potential to contribute to community healthcare and fight against COVID-19.

## 1 Introduction

The COVID-19 pandemic, caused by the novel coronavirus SARS-CoV-2, was first reported in Wuhan, China, in December 2019. The disease subsequently spread to multiple countries, resulting in sustained community transmission worldwide (1). The World Health Organization (WHO) declared COVID-19 a pandemic on March 2020 (2). Early public health responses focused on non-pharmaceutical interventions and measures at individual, community, and national levels to limit the spread of the infection due to the absence of medication or vaccines and the immense strain on healthcare systems (3). The rollout of the first COVID-19 vaccine in December 2020, just 1 year after the virus was identified, marked an extraordinary milestone in vaccine development and implementation. This rapid progress was a testament to the dedication of scientists, public health officials, and governments globally (4).

Despite this achievement, the vaccine rollout faced challenges such as vaccine hesitancy and supply shortages (5). The middle phase of the pandemic was characterized by a decline in the number of cases and deaths but also saw the continued circulation of SARS-CoV-2 and the emergence of new variants (6, 7). This phase presented challenges, including balancing public health measures with economic and social needs and sustaining vaccination efforts to ensure adequate population coverage (8). Governments used this time to invest in public health infrastructure, including testing and contact tracing systems, while supporting economic recovery and social resilience (9).

Although the COVID-19 pandemic continues, the situation has improved considerably since its early days, owing to several factors. These include the widespread availability of vaccines and boosters, the development of effective treatments, and increased awareness of preventive measures such as wearing masks in public indoor settings, social distancing, and frequent handwashing (10). However, the emergence of new variants such as BA.2.86, EG.5, and HK.3 underscores the ongoing nature of the pandemic. These variants are associated with mutations that may enhance their transmissibility and ability to evade immunity conferred by prior infections or vaccination (11).

EG.5, first detected in February 2023, has become the dominant variant in several countries, including the United States. Its mutations enable it to escape antibodies generated from previous infections or vaccinations. Despite its increased transmissibility, EG.5 does not appear to cause more severe illness than other Omicron subvariants. BA.2.86, first reported in Denmark in June 2023, has spread to over 20 countries. Preliminary studies suggest it may be more transmissible than other Omicron variants, with mutations conferring enhanced immune escape. Similarly, HK.3, identified in Hong Kong in August 2023, exhibits limited data on its transmissibility and immune evasion potential, warranting further research.

The emergence of these subvariants underscores the importance of remaining vigilant and adhering to preventive measures, including immunization, mask-wearing, and social distancing, to mitigate the risk of infection. This study emphasizes the role of advanced technologies such as the Internet of Things (IoT) and machine learning (ML) in addressing the challenges posed by the ongoing pandemic. IoT, a technology enabling connected devices to collect and transmit data autonomously, has gained significant attention in healthcare applications.

This work proposes a smart entry gateway system comprising multiple subsystems, including face mask recognition, face shield detection, sanitization systems, temperature monitoring systems, and vaccine verification. The system leverages a hybrid object classification model combining MobileNetV2 and VGG19 for face shield detection, achieving robust and accurate results. A CoWIN-like database has been developed for vaccine certificate authentication, adding an extra layer of safety. The face mask detection module incorporates advanced image augmentation techniques and a hybrid model, enhancing accuracy by reducing redundant features and capturing critical facial characteristics. These innovations ensure precise detection of masks and shields while minimizing computational complexity.

The contributions of this study are summarized as follows:

1. **Holistic Entrance Gateway System Integration**: The proposed system integrates multiple subsystems into a unified gateway, enabling comprehensive screening of entrants at a centralized point.
2. **Hybrid Model for Face Shield Detection**: The system uses a hybrid object classification model combining MobileNetV2 and VGG19, ensuring accurate detection of face shields under challenging conditions.
3. **Integrated Face Mask and Face Shield Detection**: The system effectively detects face masks, whether used alone or in combination with face shields, providing a comprehensive assessment of health risks.
4. **Vaccine Verification Database**: The system incorporates a CoWIN-like database to authenticate vaccination certificates, reducing the risk of viral transmission.
5. **Real-time Google Cloud Database**: The system leverages a real-time Google Cloud database for efficient data processing with minimal latency, ensuring up-to-date health status information.

The remainder of this paper is organized as follows: Section 2 reviews existing studies on the topic. Section 3 describes the proposed system and its implementation. Section 4 presents the results and discussion, while Section 5 concludes the work by summarizing the findings and implications.

## 2 Related work

Cui et al. (1) work delves into a decision-making method addressing the challenges posed by the dynamic nature of the novel coronavirus. This approach involves synthesizing expert opinions to formulate effective strategies for urban security, economic stability, and medical system resilience. This method expedites plan determination and optimizes expert insights by identifying experts with significant differences in opinions and iteratively modifying them through consultation. The study emphasizes the crucial role of strict entry policies, particularly robust detection and quarantine measures for international travelers, in containing the spread of the virus. While acknowledging contributions, the review highlights areas for improvement, such as refining the consensus model and adapting decision-making for large-scale contexts. Ongoing research is required to enhance the accuracy of the proposed method in the evolving landscape of COVID-19, offering valuable insights into urban resilience, economic stability, and healthcare system integrity.

Whitfield et al. (12) highlight that the interventions implemented by the logistics sector in the nascent period of the pandemic likely contributed to a reduction in the peril of workplace and community spread by safeguarding both clientele and employees. The accessibility of lateral flow tests is identified as an additional valuable protective measure that is particularly effective when combined with targeted isolation measures for high-risk contacts. The synergistic approach of these interventions underscores their potential to mitigate the transmission risks during the ongoing challenges posed by the pandemic.

Rajasekhar et al. (13) demonstrated the successful implementation of five diverse active learning approaches on a basic face mask detection dataset by employing a convolutional neural network. Notably, all models achieved commendable accuracy scores even after a brief training period of 50 epochs. Random Sampling emerged as the most effective method, attaining a maximum accuracy of 72%, followed by the Largest Margin Uncertainty at 70.23%, Smallest Margin Uncertainty at 69.89%, Entropy Reduction at 64.49%, and least confidence at 32.73%. By assigning only 25% of the dataset to the functioning learning model, this study features its fundamental ability to improve precision through independent data labeling and a decrease in unlabelled data. This study proposes that continual training enhances model occurrence, and selecting a particular query strategy, coupled with hyperparameter tuning, holds promise for emerging robust models relevant to future training and research detection.

Gautam et al. (7) effectively implemented five active knowledge approaches on an important face mask detection dataset, leveraging a convolutional neural network. Remarkably, all simulations demonstrated creditable accuracy even with a crisp training period of 50 epochs. Random Sampling had the highest accuracy at 72%, followed by the Largest Margin Ambiguity at 70.23%, Smallest Margin Uncertainty at 69.89%, Entropy Reduction at 64.49%, and least confidence at 32.73%. Allocating only 25% of the dataset to the active learning model, this study underscores its inherent capacity for autonomous data labeling and the reduction of unlabelled data to enhance accuracy. The study suggests that extended training further improves model performance, and selecting a specific query strategy, coupled with hyperparameter tuning, holds promise for developing robust models applicable to future training and research endeavors.

Shukla et al. (2) study underscores the significance of knowing and forecasting COVID-19 cases, including confirmed, death, and recovered cases, to facilitate pre-planning of effective measures. A neural network model, developed with pretreated datasets and trained on real data from government sources, predicts these cases. The model aids policymakers in understanding case patterns and implementing safety measures, even without detailed symptoms. This study emphasizes the utility of the model during pandemics and envisions its future use for forecasting epidemic situations, minimizing economic losses, and devising recovery plans. To enhance the study, future iterations could include wide-ranging end-to-end datasets and explore numerous time-series models such as RNN, BILSTM, and BERT, among others, to improve metrics such as RMSE and R-squared. Furthermore, extending the opportunity for global case prediction in a comprehensive form could broaden the applicability of this study.

Khan et al. (3) systematically reviewed a range of AI-IoT technologies, including blockchain, cloud computing, fog computing, sensing technologies, machine learning, deep learning techniques, and robots, which are instrumental in supporting COVID-19 efforts. Employing the proposed taxonomy, this study not only relies on conventional review methods but also introduces a novel approach that incorporates systems from image processing, dynamical systems, and machine learning that offer a unique understanding of specific technologies. Theoretical and practical aspects, including the superiority, drawbacks, shifts, and differentiation of these technologies, were explored. In addition, potential future work that remains underexplored is proposed. This study underscores the pivotal role of healthcare in utilizing AI-IoT technologies and concludes that the hybridization of fog computing and cloud computing in IoT, coupled with advancements in deep learning and blockchain technology, will shape the future landscape of COVID-19-related AI-IoT technologies.

Kumar et al. (4) conducted an analysis based on a dataset, leveraging a projected model with machine learning (ML) systems to yield favorable outcomes. Among the ML prediction models assessed, the proposed K-means SVM model emerged as the most effective and consistent, showing superior performance. This finding holds valuable implications for predicting the spread of pandemics/epidemics at both country-specific and global scales. This analysis faces the challenge of a growing dataset with an exponentially increasing number of cases. The amalgamation of the SVM and K-means algorithms proved particularly effective in predicting results with enhanced accuracy compared to alternative methods. Looking ahead, the incorporation of additional ML algorithms is suggested to further augment the prediction accuracy in future studies.

Shashank Reddy et al. (14) wrote about temperature monitoring using the LM35 sensor and BoltIoT. The system is connected to the BoltIoT cloud which holds instructions to detect when the temperature is within the normal range or above. It also sends out an alert through an API affiliated with Telegram and sends an SMS warning to the user and supervisor to take care of the particular individual.

Dutta et al. (15) described how the BoltIoT chip can be used for several purposes. They discuss connecting it to a PIR sensor which is used to detect motion, a cool, light sensor, a gas sensor, and a range of several different features and how they can be combined using the BoltIoT microprocessor chip.

Stolojescu-Crisan et al. (10) presented a brilliant comparative analysis of communication protocols that can be used with various controllers or processors, their UI, and their applications. They also presented a comparative study of several open-source home-based automation programs, a few of which can also be used with the proposed system.

Naufal et al. (16) proposed a comparative study of image classification which involved a practical analysis using K nearest neighbors (KNN), support vector machine (SVM), and convolutional neural network (CNN). CNN performed the best out of all three with an average precision of 96% and an average testing time of 2507.802s for testing.

A juxtaposition of YOLOv3, YOLOv5, and MobileNet-SSD for real-time recognition was proposed by Iyer et al. (17) YOLOv3 uses only one neural network, which contributes to real-time inputs. YOLOv2 uses 30 convolutional layers of architecture due to which the data loses its fine-grained features and hence is not suitable for small object detections. YOLOv5 introduced Python as the language used for the algorithm, unlike previous versions which used C++. They proposed a network of three parts: –the backbone, neck, and head. The A backbone consists of a CNN layer, the neck consists of a set of layers to combine image features, and the head consists of features to perform localization and classification. MobileNet is used when the models are more complex and there are limitations on the GPU used. It was designed specifically for mobile and embedded applications at high speeds.

Dilu et al. (18) put forward a temperature monitoring system using an MLX90614 sensor. When a person enters any premises, the system measures the body temperature of a person utilizing an IR temperature sensor MLX90614. The MLX90614 temperature sensor provides a 17-bit digital value using the I2C protocol. The system asks for the name and phone number of each person by using a speech-to-text converter and then it is stored in the database. It makes use of Raspberry Pi model B for storing the collected data. It will print the text on a 16*2 LCD* using a wireless Bluetooth medium. So, to receive the data sent through the mobile phone, it is necessary to interface the Raspberry Pi and 16*2 LCD* with Bluetooth. The sanitization system is interlinked with the BoltIoT protocol, which aids the logical transition of the IR sensor interlinked with the relay module for pumping the sanitizer. Therefore, the proposed work has the potential to make a significant contribution to public health and the fight against COVID-19.

## 3 Proposed methodology

In this study, we introduced a lightweight face mask recognition model. developed and deployed using a hybrid approach that combines MobileNetV2 and VGG19. The MobileNet model is a net model that utilizes depth-wise separable convolutions (19–21). Its depth-wise separable convolution has two layers: depth-wise and point convolutions. It is based on an inverted residual structure, in which the outstanding connections are amongst the bottleneck layers. On the other hand, the VGG19 model uses a more traditional deep convolutional architecture with 19 layers, emphasizing very small (3 x 3) convolution filters to capture detailed features. By combining the efficiency of MobileNetV2 with the fine-grained feature extraction of VGG19, our hybrid model delivers improved performance in face mask recognition.

MobileNetV2 is a convolutional neural network architecture that works on mobile platforms. It is built on a reversed residual structure, in which the residual links are between the bottleneck layers. The intermediate expansion layer applies a lightweight depth-wise convolution to separate features as a source of nonlinearity. Overall, the architecture of MobileNetV2 contains an initial fully convolutional layer with 32 filters, followed by 19 residual bottleneck layers.” In our proposed work, we use a hybrid model that combines MobileNetV2 and VGG19. This approach is beneficial because, unlike other algorithms like YOLO or VGG19 on its own, it doesn't require a high-end GPU and can run efficiently on standard computers without overwhelming the RAM. MobileNetV2, with its faster training time compared to traditional CNNs, helps us achieve our goal of reducing the time needed for model training.

MobileNetV2 takes less time to train than a CNN (22) gives a boost to our research, as we aim to reduce the time required as much as possible. Many face mask detection models have been developed (23–27); however, most of them have been intended to increase recognition accuracy (8, 28, 29). However, the obligation of minimizing computational difficulty is generally ignored. To attain real-time performance, the face mask detectors must operate on a high-end GPU (30–33). In our hybrid approach, combining MobileNetV2 with VGG19, we aim to balance computational efficiency with high accuracy, making it feasible for deployment even on standard computers.

**Figure 1:**
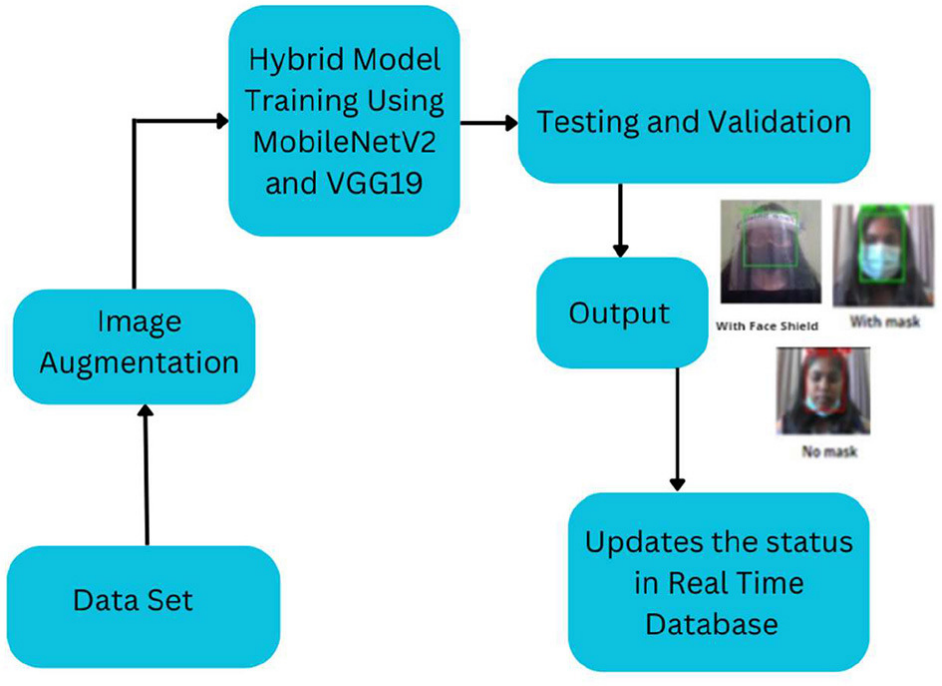
Workflow diagram of the hybrid face mask and face shield detection model.

Figure 1 shows the flow of the face-mask and face-shield detection process. The program starts by creating a list of images in our dataset directory and then using the list of data and classes. Then, we generate arrays to append all image data and label them with or without a mask. The whole dataset was divided into testing and training sets and the labels were stratified to avoid regression. Here, 80% of the dataset was used for training and 20% of the dataset was used for testing. The machine learning library Sklearn was used to preprocess the data. The values of the preliminary learning rate, number of epochs to train, and batch size were initialized.

In this work the process is trained using a diverse dataset of 6,704 images of faces without masks, capturing a range of skin tones, angles, and conditions like occlusion. The dataset also includes images of faces with masks, including those partially covering the face or being held by hands, which adds variety and strengthens the model's robustness. We also included 955 images of faces with face shields to further diversify the dataset. Although our dataset is relatively small, our hybrid model makes the most of it by leveraging advanced techniques to achieve high accuracy even with limited data. Additionally, we incorporated functionality to detect masks that aren't worn properly, ensuring our model can handle a wide range of scenarios. To maximize the effectiveness of our smaller dataset, we used image augmentation techniques to artificially expand it. By applying methods like shearing, contrast adjustment, horizontal flipping, rotation, zooming, and blurring, we were able to enhance the training images and introduce a variety of Gaussian variations. This careful design and augmentation process allows our model to achieve high accuracy, despite using less data.

To evaluate the performance of the hybrid model, we need to measure the accuracy, precision, F1 score, and recall, which can be found using equations (1) to (4) as given here.


(1) $$\begin{array}{l} Precision=TP/\left(TP+FP\right) \end{array}$$

(2) $$\begin{array}{l} Recall=TP/\left(TP+FN\right) \end{array}$$

(3) $$\begin{array}{l} Accuracy=\left(TP+TN\right)/\left[\left(TP+FP\right)+\left(TN+FN\right)\right] \end{array}$$

(4) $$\begin{array}{l} Fl\;score=2\left[\left(Precision*Recall\right)/\left(Precision+Recall\right)\right] \end{array}$$

Here, “True Positive” is “TP”, “True Negative” is “TN”, “False Positive” is “FP,” and “False Negative” is “FN.”

### 3.1 Dataset and augmentation

The dataset for the hybrid model in the smart gateway system consists of selected images designed for accurate face mask and face shield detection. The data is organized into training, testing, and validation sets to build a solid and reliable foundation for developing and evaluating the model. The dataset is a collection of 6,704 images of faces without masks, taking a varied range of features such as skin tones, facial angles, and various conditions like sealing. This diversity ensures that the model is visible to a wide variety of real-world circumstances, enhancing its capability to take a broad view across diverse individuals and atmospheres.

In addition to the unmasked faces, the dataset includes images of faces with masks, surrounding various scenarios where the masks are partly covering the face or being held by hands. This addition enhances another layer of complexity and realism to the training process, allowing the model to identify masks well even when they are not worn properly.

Furthermore, the dataset features 955 images of faces with face shields, which diversifies the training data and reinforces the model's capability to precisely detect protective gear. This diversity is critical for ensuring that the model can efficiently distinguish between masks and face shields, as well as handle situations where multiple types of defensive equipment are present.

Given the comparatively small size of the dataset, advanced image augmentation methods were employed to precisely expand it. These approaches include trimming, contrast change, parallel flipping, rotation, zooming, and blurring, which encompass a wide range of Gaussian variations. By enhancing the training images through these extensions, the dataset efficiently mimics a larger and more varied set of real-world conditions.

The augmentation process was carefully designed to make the most of the dataset, helping the hybrid model achieve high accuracy despite the limited data. This ensures the model can detect face masks and shields not only in ideal conditions but also when masks are worn incorrectly, improving its ability to handle different situations. Overall, this dataset provides a solid foundation for training a model proficient in accurate face mask and face shield detection, with the possibility to generalize well across several real-world applications. A hold-out validation approach was used to ensure the statistical reliability of the model's performance.

The metadata for each image includes the following information: Filename: Unique identifier for each image. Class Label: Indicates whether the image is of a masked face, an unmasked face, or a face with a shield. Resolution: All images have a resolution of 224 x 224 pixels. Source: Indicates the origin of the image (e.g., dataset name or manual collection).

Before using the images for training and evaluation, several preprocessing steps were applied to ensure consistency and enhance the model's performance:

1. Resizing: All images were resized to 224 x 224 pixels.
2. Normalization: Pixel values were normalized to a range of [0, 1].
3. Augmentation: To increase the robustness of the model, various augmentation techniques were applied, including rotation, flipping, and color jitter.

We use a hybrid model that combines MobileNetV2 and VGG19 to accurately detect face masks and shields in images or videos. This model blends the speed and efficiency of MobileNetV2 with the detailed feature recognition of VGG19, creating a powerful tool for real-time detection. To train the model for face shield detection, we put together a dataset of both positive and negative images. The positive images feature people wearing face shields, helping the model learn to recognize them accurately. The negative images, in contrast, don't have face shields but are otherwise similar to the positive ones, allowing the model to distinguish between the two scenarios effectively. By training on these datasets, our hybrid model becomes adept at identifying whether a face shield is present or not. Unlike traditional methods like the Haar Cascade algorithm, our deep learning-based approach provides more precise and reliable results. Once the model is trained, it's deployed using OpenCV to process images in real time, successfully identifying and marking faces with masks and shields. This advanced method not only improves detection accuracy but also runs efficiently on standard hardware, making it practical for everyday use.

The next important step in our program is accessing the camera, which we do using OpenCV. Once the camera captures a frame, we convert it to grayscale. This simplifies the image by reducing it to two dimensions, ensuring that different colors in the image don't interfere with detecting face masks and shields. After capturing the frame, we run it through our hybrid model using the predict() function. The model analyzes the frame to check for face masks or shields and identifies their location by creating a bounding box around them. The bounding box includes the x and y positions, as well as the height and width of the detected object. We then use OpenCV to draw these bounding boxes on the frame, clearly highlighting the detected face masks or shields. This process happens in real-time, enabling continuous and immediate detection.

#### 3.1.1 Data preprocessing

All images were normalized to a pixel range of [0,1][0,1][0,1] using the rescale = 1./255 parameter from TensorFlow's ImageDataGenerator. Images were loaded in batches via the flow_from_directory() method, with class_mode = “categorical” to enable one-hot encoding of the class labels. This setup ensured an efficient and consistent data pipeline during both training and evaluation phases. To meet the input requirements of both MobileNetV2 and VGG19, each image was resized uniformly to 128 × 128 pixels.

#### 3.1.2 Hybrid feature extraction model

The proposed detection model leverages a hybrid architecture that integrates MobileNetV2 and VGG19, combining the strengths of both networks for more robust feature extraction:

- MobileNetV2 is a lightweight CNN designed for efficient performance on mobile and edge devices. It contributes to rapid feature extraction using depth-wise separable convolutions.
- VGG19, a deeper and more expressive network, aids in capturing fine-grained features thanks to its deeper convolutional stack.

Both models were initialized with pre-trained ImageNet weights, and their classification heads were removed (include_top = False). A shared input layer with shape (128, 128, 3) was used to feed both networks.

The outputs from MobileNetV2 and VGG19 were each passed through a Global Average Pooling layer (pool size of 4 × 4), followed by flattening. These two feature vectors were then concatenated to form a unified representation. This combined feature vector was input to the classification head, which consisted of:

- Dense(256, activation = ‘relu')
- Dropout(0.5)
- Dense(3, activation = ‘softmax') for classifying into three categories.

To retain the learned features and avoid overfitting, all layers in both base models were frozen during training.

#### 3.1.3 Training and hyperparameter settings

The model was compiled using the Adam optimizer with a learning rate of 0.0002, and categorical cross-entropy was used as the loss function. The training was carried out for 10 epochs with a batch size of 32. The Table 1 below summarizes the key hyperparameters:

**Table 1:** Training configuration and hyperparameters used for model development.

**Parameter**	**Value**
Optimizer	Adam
Learning rate	0.0002
Loss function	Categorical cross entropy
Batch size	32
Epochs	10
Metrics	Accuracy
Base layers frozen	Yes (All)

To mitigate overfitting, the following callbacks were implemented:

- Early Stopping to monitor both training and validation accuracy, with patience values set to 8 and 2, respectively.
- Model Checkpoint to save the best-performing model weights based on validation performance.

#### 3.1.4 Evaluation and metrics

The final model was evaluated on a separate test set containing samples from all three classes. Predictions were generated using the model. Predict(), and performance were assessed using scikit-learn's classification report.

The classification results were reported using the following metrics:

- Precision
- Recall (Sensitivity)
- F1-Score
- Accuracy
- Class-wise Accuracy

Additionally, per-class accuracy was manually computed to provide a deeper understanding of the model's performance on each individual category.

### 3.2 Statistical validation of the hybrid model

To evaluate the robustness and reliability of our hybrid face mask and face shield detection model, we conducted a comprehensive analysis using standard classification metrics on an independent test set covering all three classes: face (no mask), masked, and shield. The results are presented in Table 2.

**Table 2:** Classification performance of the proposed hybrid model across face mask and face shield categories on the independent test dataset.

**Class**	**Precision**	**Recall (Sensitivity)**	**F1-Score**	**Support**
Face	1.00	0.97	0.98	2,000
Masked	0.94	0.93	0.94	427
Shield	0.83	0.99	0.90	305
Macro Avg	0.92	0.96	0.94	2,732
Weighted Avg	0.97	0.97	0.97	2,732

The overall accuracy across all classes was **97%**, with a macro-averaged F1-score of **94%**, reflecting balanced performance across imbalanced class distributions. Notably, the **shield class** achieved a **99% recall**, ensuring that face shield detection is highly sensitive with minimal false negatives — a critical requirement in public safety applications.

These results confirm that the proposed model performs reliably across diverse scenarios. The combination of **MobileNetV2** and **VGG19** not only enables high precision for standard mask detection but also achieves strong generalization even for underrepresented classes like face shields.

### 3.3 Tkinter GUI

The Tkinter GUI is a Python library used to make user interfaces with Python. This is the quickest and simplest method for GUI applications. We use this library for the particular use case because it makes the application lighter and more user-friendly as the file size becomes very small (34). With Tkinter, the usability of code increases, and the reusability of code doubles. In addition, it also makes use of much less disc space compared to any Python server. UIs made in (HyperText Markup Language) HTML have to make use of (Cascading Style Sheets) CSS for styling purposes and JavaScript for storing and retrieving data from the user, and that data would be interlinked with HTML in a JavaScript Object Notation (JSON) file (35). Thus, when the program is run, it introduces a certain amount of latency to the entire application. Instead of HTML, when we use Tkinter, we work on a Python framework on which we can develop the UI and carry out our application development. This causes the file to be completed without lagging or with negligible latency.

### 3.4 Database management

When the user first enters their data into the system through the Tkinter GUI, the form accepts the data, it stores all the information in the form of an array. This array has now been added to the firebase using Python. This provides us with two benefits. Instead of scraping the information from JavaScript, then making a query, forming the documentation, and adding it to the structured query language (SQL) database, which is run on the local server. Increasing the latency, we scraped the data in real-time and directly uploaded the data to a Google Firebase real-time NoSQL database, enabling seamless integration and efficient data synchronization with the Python-based application. To integrate the firebase, we need a JSON certification. This certification represents the connection between the Google e-console and the Python algorithm that we developed. By scraping the data and uploading it to the firebase, we integrated our online real-time database with the Python cloud, This integration supports real-time updates to user health records while minimizing latency, making it suitable for responsive decision-making at entry checkpoints. To integrate the firebase, we need to instantiate an application with the credential data certification of the JSON file. After scraping the data, we set a default class, following which we connected our database in real-time through the Hypertext Transfer Protocol Secure (HTTPS) port. HTTPS is the safe adaptation of HTTP, which is the essential protocol used to send information between an Internet browser and a site. HTTPS is encrypted to build security for transmitting the information. This is especially significant when clients send confidential information, for example, by signing a bank account, email administration, or a medical insurance provider. Now, we use the update, set, and create methods. Once the user clicks the Submit button, the values are updated in the database. It updates the user key in the dictionary of the JSON values, that is, when the form is submitted, all the data are stored in the form of a list. Because we are working in real-time, we need to make use of JSON to extract, store, and use the data that is being submitted by the user; hence, we convert the list first into JSON, and then upload the JSON file to our database and convert it into a dictionary form as the firebase can only comprehend information if it is present in the form of a dictionary. To have a reference to call the respective person's database, we run a for loop to form a list of the IDs of all personnel. This user ID will be the key by which a person's database will be called to edit or update. Now, whenever there is a need to update any of their details, such as their vaccination status or the daily screening tests, the main ID key will be called out, through which the whole database of the person will become available to the admin.

### 3.5 QR generation module

QR codes were generated using the PyQRCode Python library (also referred to as Pyqrgen), which automates the process of creating scannable codes efficiently. Each QR code encodes employee-specific metadata, including:

- Name
- Employee ID
- Vaccination status
- Certificate ID
- Last dose date

The generated QR codes are saved locally as PNG images using the png module. PNG was selected due to its efficient compression and minimal storage footprint, making it ideal for storing identification data. By keeping the QR codes on local systems instead of uploading them to a cloud database, we reduce storage demands and prevent latency in real-time operations. Employees receive their QR codes once, which they retain and use when required.

### 3.6 Timestamp logging and data update mechanism

When an employee submits their details, the system logs the timestamp using Python's time library. The strftime() function records the exact date and time, while time.localtime() converts epoch time into the local time zone. This aids in aligning updates with regional settings and helps manage execution timing between code cycles.

The system ensures that any new information or updates to an employee's profile overwrite the existing data, avoiding redundant database entries and maintaining a clean, up-to-date record.

Real-Time QR Verification System

At entry points, real-time verification is carried out using the following components:

- OpenCV for capturing live video frames
- Pyzbar for decoding QR codes from those frames

Once a QR is decoded, its contents are securely cross-checked with a Firebase NoSQL database using HTTPS-secured API calls. The system verifies the employee's vaccination validity and simultaneously logs their entry time and temperature reading.

### 3.7 Database security and privacy measures

To protect data and uphold user privacy, the following security strategies were implemented:

Table 3 illustrates the security and privacy measures integrated into the system architecture. Though biometric or multi-factor authentication is not currently implemented, the system uses Google Cloud Console's service roles for role-based access control. The QR codes serve as a lightweight, one-time authentication token, ensuring secure and efficient verification without long-term personal data retention.

**Table 3:** Security and privacy measures implemented in the system architecture.

**Component**	**Implementation**
Data transmission	Encrypted via HTTPS for secure communication
Access control	Admin-only access enforced using Firebase Authentication
Data storage	QR codes are stored locally, avoiding cloud-based persistence
Realtime database	Access protected using IAM roles and API tokens via Firebase
QR code content	Encoded with only essential data—excludes sensitive personal information
User privacy	No storage of phone numbers, emails, or personally identifiable information (PII)
Audit trail	Only entry time and temperature logs are recorded for security auditing

The proposed QR-based verification system is designed to strike a balance between real-time operational performance and adherence to fundamental data protection principles. By storing QR codes locally on the employee's device and performing identity and vaccination verification through secure, API-based communication, the system significantly reduces reliance on cloud storage and limits data exposure.

Access to the Firebase backend is controlled using role-based permissions, and all data exchanges are encrypted using HTTPS, ensuring secure transmission. Although the system does not formally comply with HIPAA or GDPR standards, it adopts key best practices commonly used in embedded healthcare-related applications. These include minimizing the collection of personally identifiable information (PII), avoiding persistent tracking of individuals, and securing all interactions throughout the verification process.

### 3.8 Temperature sensing module

A “normal” body temperature can lie within a range, of 97°F to 99°F. We used an Arduino Uno Board, Arduino IDE, and MLX90614 digital non-contact infrared thermometer sensors for the temperature monitoring system. The IR temperature sensor (MLX90614) was interfaced with the Arduino Uno Board using a breadboard and a few jumper wires which connected the input/output and power pins of the Arduino to the respective pins of the sensor.

**Figure 2:**
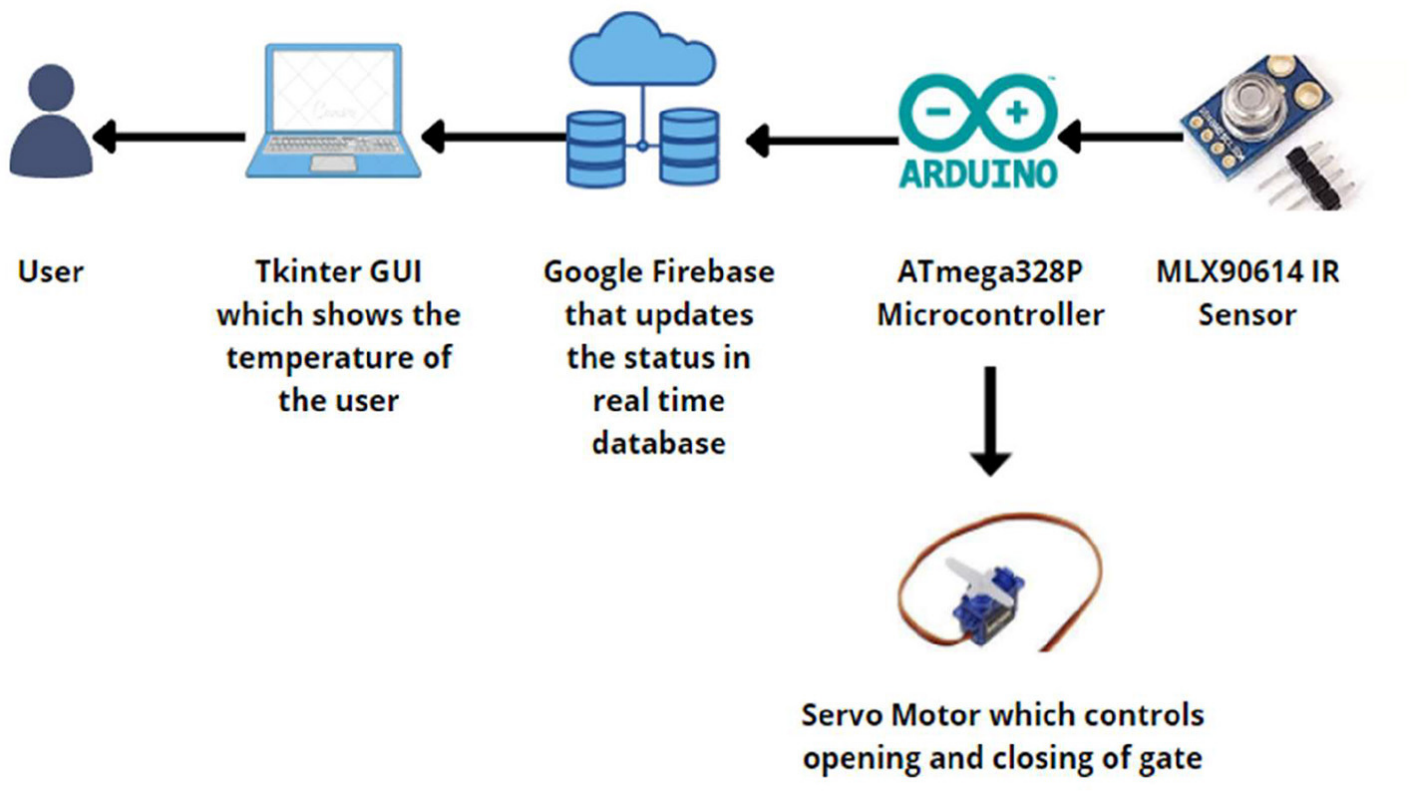
**System** functional overview of the contactless temperature monitoring system showing the process of capturing and logging body temperature using the MLX90614 infrared sensor and Arduino Uno microcontroller.

As shown in Figure 2, while running the system, the sensor extracts the temperature of the individual and sends it to the database to record the data. If the temperature falls outside the normal temperature range, an alert is sent to the supervisor to take appropriate action, and the individual is not allowed to enter the premises of the institution. If no anomaly is found in the recorded temperature, the system simply grants the user access to the next screening step, which is the oxygen level monitoring and heartbeat monitoring system.

### 3.9 Sanitization module

**Figure 3:**
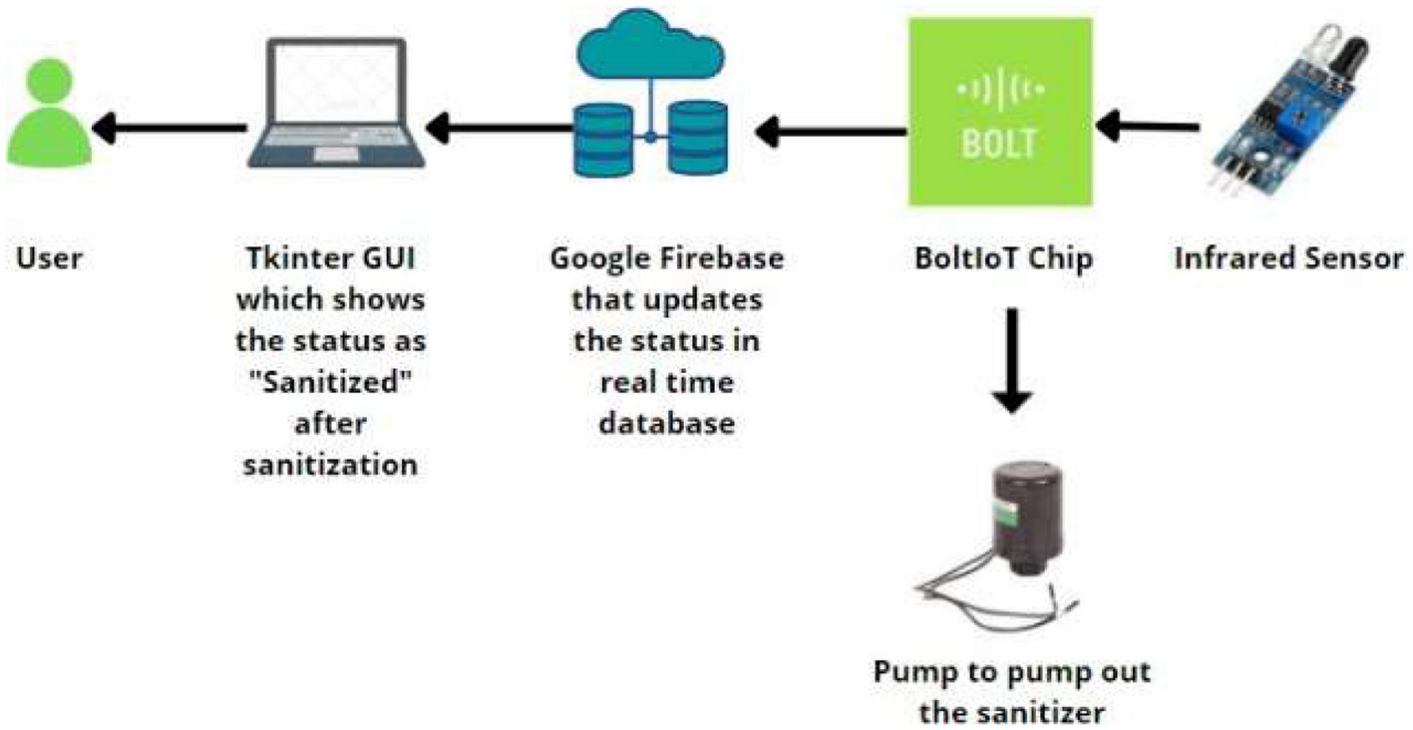
Workflow of the automated hand sanitization module utilizing an IR sensor to detect proximity, which activates a relay-controlled pump to dispense sanitizer.

The employee must be sanitized before entering the organization, as it is mandatory as a precautionary measure. To create this system, we used an IR sensor, a servo motor, and an Arduino, as shown in Figure 3.

When employees pass the face shield screening test, they must sanitize themselves. An IR sensor was used for this purpose. Because our bodies emit a good amount of heat, this heat energy is taken as infrared radiation and is captured by the infrared sensor when our hands fall within this range. Once the infrared rays are recognized, the sensor sends a message back to the relay module which pumps the sanitizer into the person's hands.

## 4 Results and discussions

### 4.1 Procedure of the face mask and face shield detection system using the hybrid model

**Figure 4:**
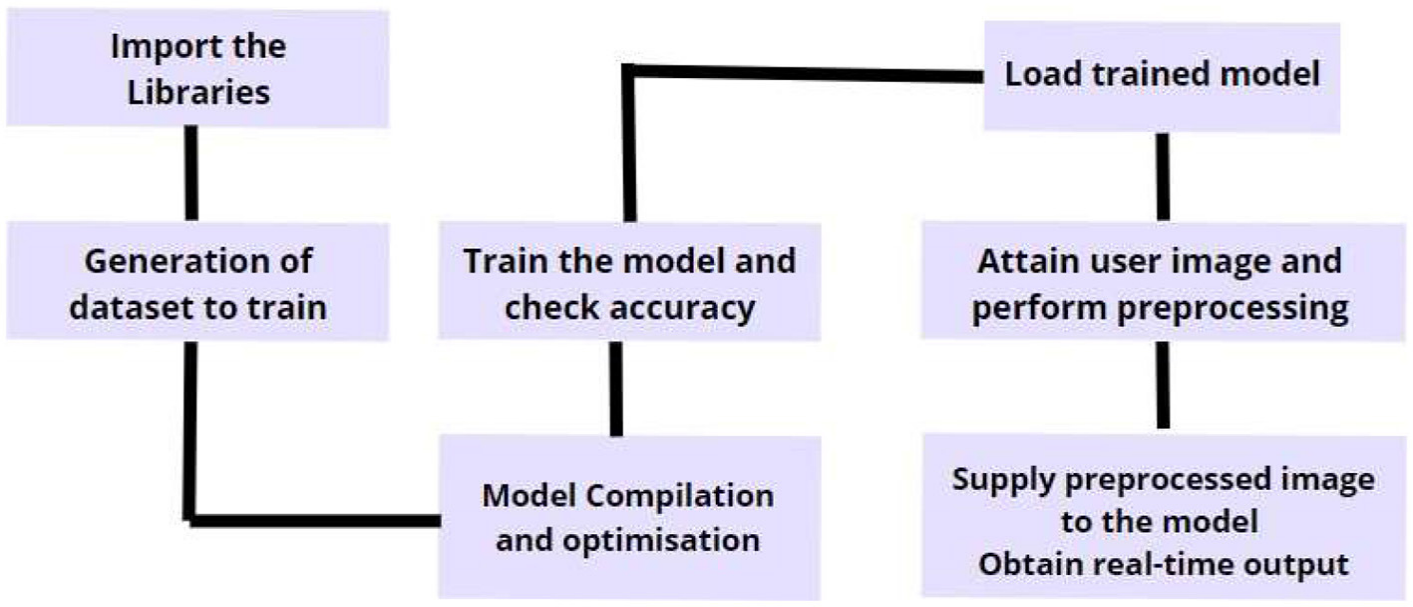
Comprehensive workflow of the integrated smart entry gateway system.

The process starts by loading the dataset and capturing images from the camera or input device, as shown in Figure 4. We set up the necessary files using system utilities and directory paths, and then process the data into binary tensors. The image labels are encoded using one-hot encoding, which prepares them for training. Next, we split the dataset into training and testing sets, ensuring that the labels are evenly distributed. To make the most of our dataset, we apply data augmentation techniques that add variety by altering the images in different ways. These enhanced images are then fed into our hybrid model, which combines the strengths of MobileNetV2 and VGG19. MobileNetV2 handles the overall structure of the images efficiently, while VGG19 digs deeper into the details, extracting key features. The model downscales the images using average pooling, and the data is reshaped to fit the required dimensions for processing. We validate these tensors using activation functions with set biases to ensure accuracy. To prevent the model from overfitting, we apply a dropout layer, and the final classification is done using a SoftMax activation function. The model is trained using cross-entropy loss with the Adam optimizer, which helps it learn effectively. Once training is complete, we use OpenCV to detect and draw bounding boxes around faces with masks or shields. These boxes are then fine-tuned to identify facial landmarks, allowing us to accurately detect the presence of masks or shields. Finally, we get confidence scores that tell us how sure the model is about each detection.

The process starts by loading sets of positive and negative images using a data generator. These images are then fed into our hybrid model, which combines MobileNetV2 and VGG19, to detect face masks and shields. The model learns to recognize these objects by identifying and marking their locations with bounding boxes. To get the best performance, we fine-tune various settings like the number of training stages, buffer sizes, and processing threads. Once the model is trained, we use it in Python to analyze live video frames captured by the camera. The model then quickly detects and classifies face masks and shields in real-time. We continuously adjust the model's settings to improve its accuracy and make sure the bounding boxes are placed correctly. The results, including how confident the model is in each detection, are then linked to a Firebase database for storage and further analysis.

### 4.2 QR code authentication algorithm

This algorithm utilizes the Open Computer Vision library that supports the input feed video stream along with the Pyzbar library for decoding the QR code. The decoded message was verified against the employee database deployed on the real-time NoSQL framework by Google Console. Upon verifications, a standardized graphical user interface developed by n Tkinter, that depicts the status of the employee or rectification updates transferred to the administration.

### 4.3 Vaccination certificate verification algorithm

This feature utilizes the Open Computer Vision library that supports input feed video stream along with the Pyzbar library for decoding the QR code. When a QR code is scanned, it extracts key particulars like the vaccination site, last date of vaccination, vaccination status, and contact number.

To verify the genuineness of the certificate, the decoded data is cross-checked against a confirmed database, such as CoWIN or a member database, hosted on a real-time NoSQL podium using Google Consoles. This referencing ensures the data matches the official archives, collateral that the certificate is genuine. If any particulars, such as vaccination dates or sites, don't match, the system flags the credential as possibly fraudulent. Upon verification, a standardized graphical user interface developed by Tkinter depicted the status of the employee or rectification updates transferred to the administration.

### 4.4 Sanitization system circuit

**Figure 5:**
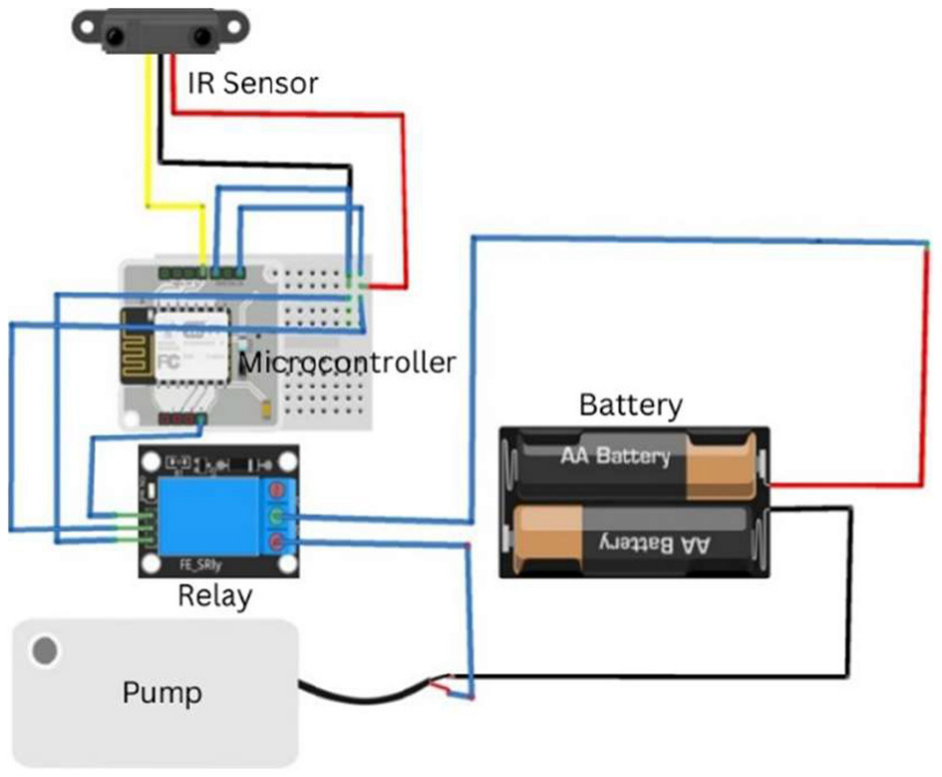
Circuit diagram of the contactless sa nitization module, highlighting the connections between the IR sensor, Arduino Uno, relay module, and pump mechanism powered by a 9V supply.

This quadrant of the system utilizes a BoltIoT chip with numerous subordinate devices. The IoT chip is interlinked with the BoltIoT Cloud and Google Firebase for real-time updates upon user usage, as shown in Figure 5. This system was implemented using an infrared sensor for the detection of human intervention and a pump connected with a 9V battery parsing through a relay module to maintain the connectivity of the circuit.

### 4.5 Temperature monitoring system circuit

**Figure 6:**
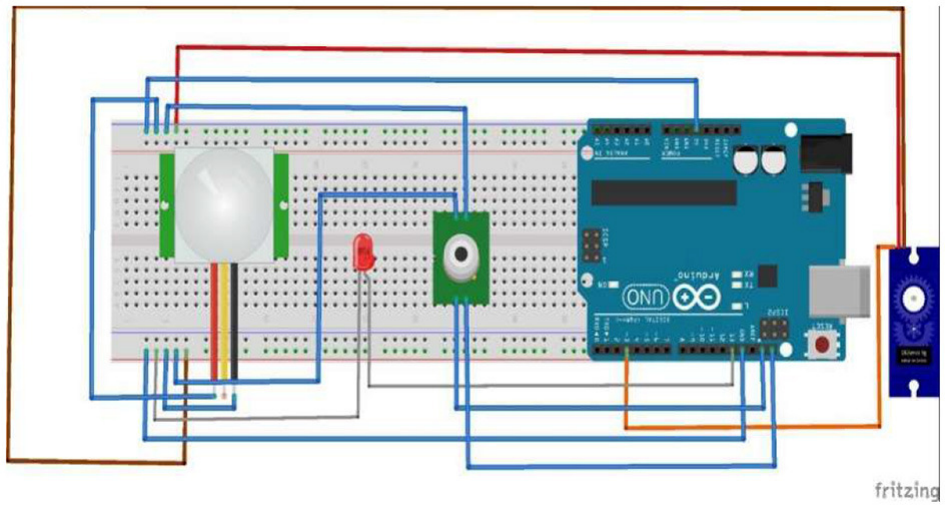
**Hardware Setup** of the temperature monitoring system, illustrating the integration of the MLX90614 sensor with the Arduino Uno for contactless thermal screening.

This component of our system is developed on the MLX90614 infrared sensor that provides ±0.5°c accuracy as shown in Figure 6, this sensor was integrated with the arduino uno board to compute the body temperature of the employee. The ATmega328P microcontroller is used for interfacing the servo motor, PIR sensor, and LED indicator for the seamless transition of our management system.

**Figure 7:**
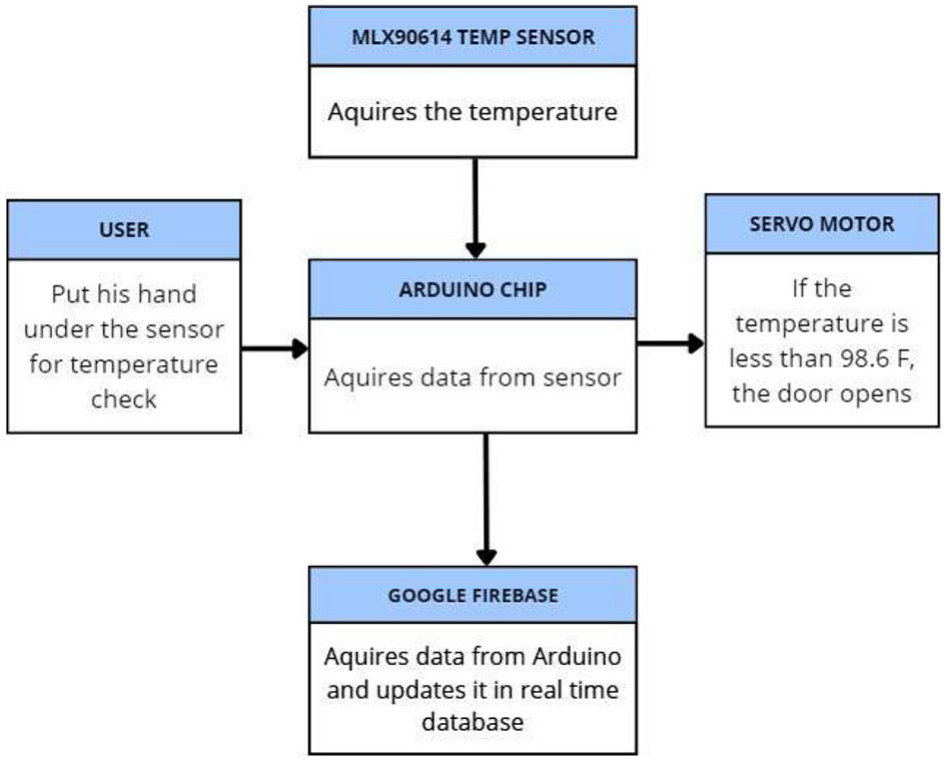
**Logical workflow**, the sequence of temperature acquisition, threshold validation, and access control for individuals entering the premises.

**Figure 8:**
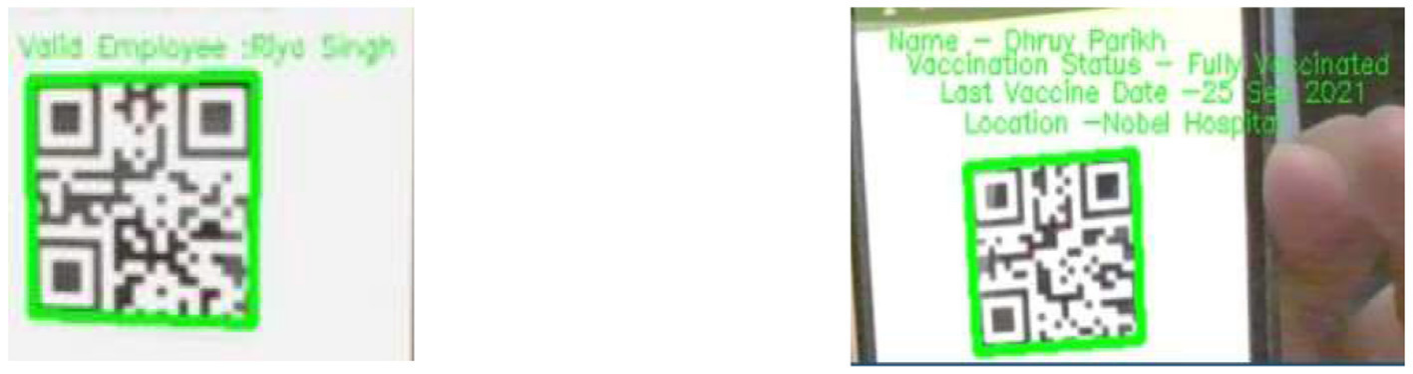
Sample QR Code used in the system, containing encoded vaccination metadata such as employee ID, vaccination status, and last dose date for authentication.

The logical operation supported by the microcontroller aids in monitoring the temperature threshold. This provides access to the entrance under ideal conditions, as shown in Figure 7. We constructed a complex apparatus that incorporates IoT and machine learning to solve the current and ongoing problem of segregating potentially infected people from non-infected people in communal spaces. The device is a camera connected to read the visual input, the QR code. The QR code, which is smaller than the rest and capable of holding larger data, is linked to each individual uniquely and can be used to establish the identity of each individual and hence authenticate their entry. Once the input is obtained, the Python libraries Zbar and Encode decode the data, and a person's vaccination and authentication details are fetched, as shown in Figure 8.

**Figure 9:**
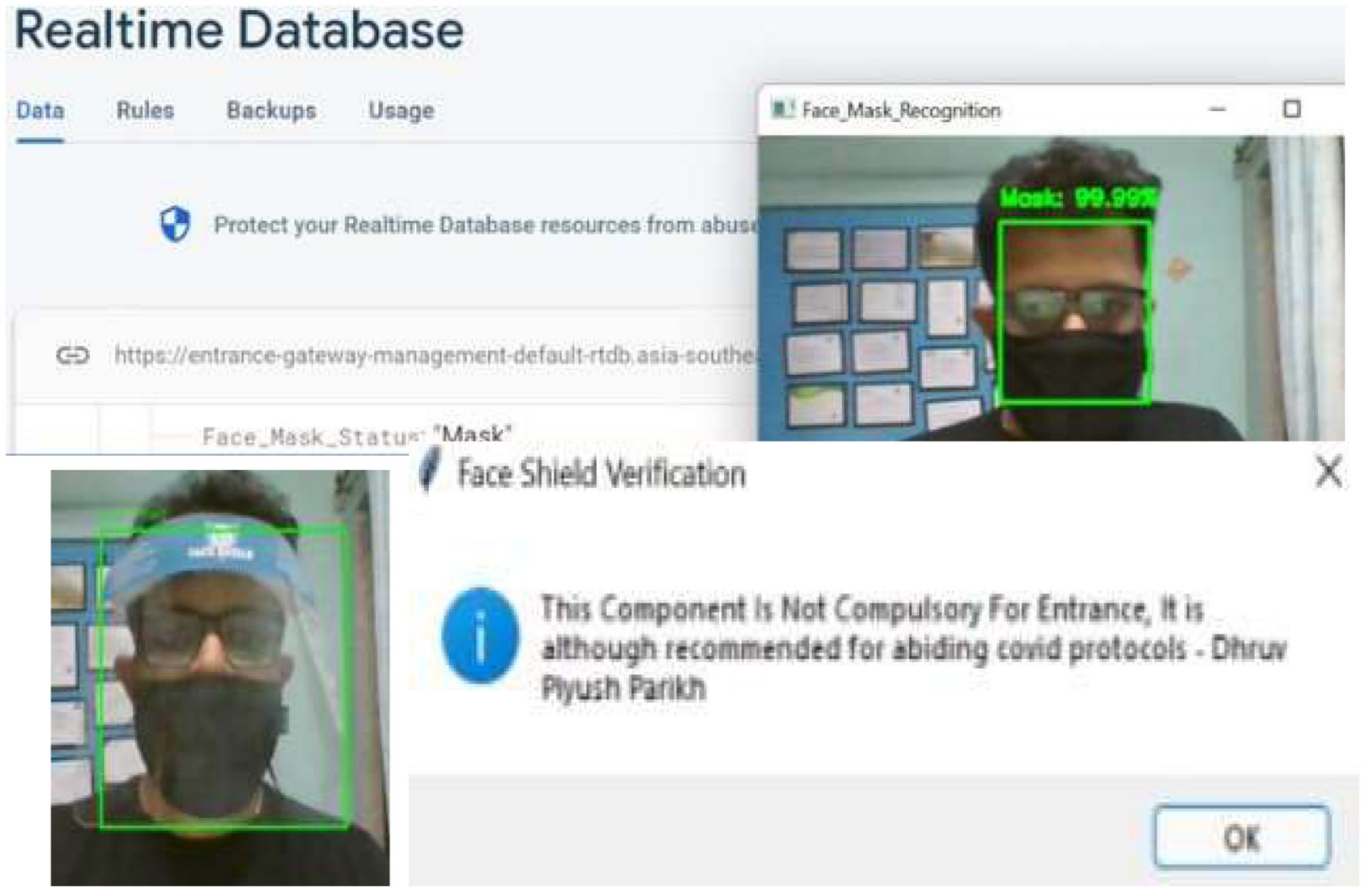
Real-Time detection output of the face mask and face shield detection module showing bounding boxes around detected protective gear using the hybrid CNN model.

After being verified as fully vaccinated and a permission entry, a face mask, as shown in Figure 9, and a face shield are detected.

Subsequently, a temperature check was performed to minimize the risk of contagion through early detection. If the temperature detected by the MLX90614 sensor is <98.6 °F then the person is allowed to enter and a message is sent to the monitoring authority/record. If the body temperature is high, an alert is sent to the records, and the person is denied entry. Similarly, if the person is not authorized, he will be denied entry, and his temperature will not be checked.

This automated system is optimized for efficiency in terms of both space and time, making it a practical solution for contactless entry checking and COVID-19 detection. The system has a small margin of error, which is more due to the varied manifestations of the disease rather than the system's performance. To further validate the system's effectiveness, we have included detailed performance metrics and comparisons with existing COVID-19 prevention systems, as discussed below.

Our results show that the face mask detection accuracy was 97%, while face shield detection improved to 99% after optimizing the model. The temperature monitoring system demonstrated high accuracy, with a minimal margin of error.

Thus, optimizing the system is more economical in terms of both space and time. We believe this automated system is efficient for entry checking and COVID detection in a contactless manner with a small percentage of error due to the varied manifestations of the disease and not due to the system's performance.

Table 4 presents a comparative analysis of the proposed system with existing COVID-19 prevention systems, emphasizing the role of machine learning models in achieving the reported outcomes. While the table primarily focuses on features and performance metrics, it implicitly highlights the significance of machine learning in the proposed system. The proposed system employs a multi-phase approach with enhanced preprocessing and CNN training, leveraging advanced machine learning techniques to improve accuracy and efficiency. This contrasts with the standard CNN architectures used in Paper 1 and Paper 2, which rely on basic implementations of CNNs with Keras, TensorFlow, and OpenCV. Specifically, the proposed system utilizes MobileNetV2 for face mask detection and an advanced deep learning model for face shield detection, achieving high accuracy rates of 97% for masks and 99% for shields. These results underscore the importance of machine learning in addressing the limitations of existing systems, which either lack face shield detection or achieve lower accuracy rates.

**Table 4:** Comparative analysis proposed system with existing COVID-19 prevention systems.

**Feature/Criteria**	**Paper 1 (36)**	**Paper 2 (37)**	**Proposed System**
Model Architecture	Standard CNN with Keras and TensorFlow	Standard CNN with OpenCV and Keras	Multi-phase approach with enhanced preprocessing and CNN training. MobileNetV2 (face mask detection), Custom Deep Learning Model (face shield detection)
Data Preprocessing	Basic data augmentation and resizing	Basic data augmentation and resizing	Advanced data augmentation, preprocessing with custom dataset
Dataset	Large dataset with multiple scenarios	Kaggle dataset (4,000 images)	Custom dataset tailored for specific scenarios
Real-Time Detection	Real-time detection implemented using Keras and TensorFlow	Real-time detection implemented using OpenCV and Keras	Optimized real-time detection with reduced computational overhead
Accuracy and Performance	No face shield detection.	No face shield detection.	99% accuracy - face shield detection
	Face mask-99% accuracy; comparisons with DenseNet-121, MobileNet-V2, etc.	Face mask-98% accuracy; comparisons with DenseNet-121, MobileNet-V2, etc.	97% accuracy – face mask
Novel Contributions	Improvements in computational efficiency and accuracy	Enhanced model accuracy and processing efficiency	Introduction of novel preprocessing techniques, multi-phase CNN model training, and optimized real-time detection

The proposed system incorporates advanced data augmentation and preprocessing techniques, which are critical for training robust machine learning models. These techniques, such as custom dataset tailoring and enhanced preprocessing, are not explicitly discussed in the existing systems, further emphasizing the role of machine learning in our approach. The high accuracy rates are a direct result of the optimized machine learning models used in the proposed system. For instance, the comparison with models like DenseNet-121 and MobileNet-V2 in existing systems highlights the superiority of our approach in terms of accuracy, computational efficiency, and real-time performance. One of the common issues with existing systems is the time it takes to process each person.

Many systems handle things like QR code scanning, temperature checks, and mask detection separately, which can lead to delays, especially in busy areas. Our system, however, integrates all these steps into a single, streamlined process, reducing the time needed to process each person. With a processing speed of <1 s, our system is much faster and more efficient than others that tend to slow down when handling multiple tasks at once.

When it comes to temperature monitoring, our system is also more precise, with an accuracy of ±0.1°F. This level of precision is crucial for ensuring that only people with normal body temperatures are allowed entry, further reducing the risk of spreading the virus in communal spaces.

Additionally, our QR code authentication and vaccination verification are highly reliable, with a 98% successful scan rate and a 99% authentication success rate. This is better than other systems that might rely on manual checks or less robust digital methods. By using QR codes linked to vaccination certificates, we offer a more dependable and scalable solution for managing public safety. Overall, our system addresses the key shortcomings of existing methods, like lower accuracy, longer processing times, and incomplete safety measures. By using advanced machine learning, efficient hardware, and seamless integration, our system provides a more reliable, faster, and user-friendly solution that's well-suited for a variety of public spaces.

Table 5 summarizes the key performance metrics of the various subsystems within our integrated entrance gateway system. These metrics demonstrate the effectiveness and efficiency of the system in real-time operation, highlighting its accuracy, responsiveness, and successful integration across all components. The comparative analysis indicates that the proposed IoT and ML-based system offers a comprehensive and efficient solution for COVID-19 prevention. It outperforms existing systems in terms of feature integration and accuracy while maintaining a moderate implementation complexity and cost. By integrating face mask detection, face shield detection, temperature monitoring, sanitization, vaccine verification, and real-time data processing, the proposed system provides a robust framework for communal health safety and contributes significantly to the fight against COVID-19.

**Table 5:** Key performance metrics of the various subsystems within our integrated entrance gateway system.

**Subsystem**	**Performance Metric**	**Result**
Face mask detection	Accuracy	97%
Face shield detection	Accuracy	99%
Sanitization System	Sanitization coverage	100%
Temperature monitoring	Temperature measurement accuracy	±0.1°C
Heart rate monitoring	Heart rate measurement accuracy	±2 BPM
QR code authentication	Successful scans	98%
Vaccine verification	Authentication success rate	99%
System integration	Real-time processing latency	<1 s
Database integration (CoWIN)	Data synchronization success	100%
User interface	User input processing time	<2 s

Table 6 presents a comparative summary of the latency and resource utilization across different models, including the proposed hybrid architecture. These metrics highlight the efficiency and practical deployment capability of our model in real-time environments such as campus screening systems and automated entry gates.

**Table 6:** Benchmark results summarizing real-time inference performance of proposed and baseline models.

**Model**	**Accuracy**	**Avg inference time (ms/image)**	**FPS**	**Model size (MB)**
Proposed Hybrid	97%	90 ms	~11	~58
MobileNetV2	94%	70 ms	~14	~14
VGG19	93%	110 ms	~9	~80
DenseNet121	95%	130 ms	~7	~33
YOLOv3	90%	200+ ms	~5	~248

While deep convolutional models like VGG19 provide high-fidelity feature extraction, they are computationally intensive when used independently. Conversely, lightweight models like MobileNetV2 offer faster inference but at the cost of reduced detection robustness. The proposed hybrid model strikes an optimal balance, leveraging the detailed feature extraction of VGG19 and the speed of MobileNetV2 to achieve reliable performance with a sub-100 ms inference time.

Moreover, with a compact model size of approximately 58 MB, the system remains deployable on edge devices with limited memory. This combination of speed, accuracy, and lightweight design outperforms traditional single-network solutions, especially in applications requiring high responsiveness and precision.

Together with integrated modules for mask and shield detection, temperature monitoring, and vaccination verification, the hybrid model forms a key part of a comprehensive, low-latency system that enhances public health security with minimal resource demands.

## 5 Internal testing

To evaluate basic system functionality and usability, the authors conducted informal in-lab testing of the proposed hybrid detection and authentication framework. These internal tests were performed without involving external participants and were not part of a formal human subject research protocol.

The complete setup—comprising the webcam, face mask detection module, QR code scanner, temperature sensor, and Firebase integration—was assembled and tested in a controlled environment to simulate real-world conditions. The internal testing confirmed:

- Real-time classification latency under 1.5 s
- Successful QR scan and database verification
- Stable temperature logging and user interface operation
- No system crashes or connectivity issues during continuous runs

While no structured trials involving third-party participants were conducted, these internal evaluations provided critical insights into component integration, real-time responsiveness, and potential deployment challenges.

Human trials with third-party participants were not conducted; the model was thoroughly tested by the authors in a simulated environment. The results confirmed that the framework operates reliably in real-time conditions and is suitable for testing in an institutional set up.

## 6 Limitations and future work

The proposed system has demonstrated promising performance in face mask and shield detection, along with real-time vaccination verification. While the initial results are encouraging, there are a few aspects that warrant further exploration and enhancement to improve system robustness and scalability in diverse application settings.

### 6.1 Detection challenges

Detection accuracy may be influenced by certain environmental and visual factors, such as:

- Low-light or uneven illumination
- Partial facial occlusion due to accessories like sunglasses, scarves, or hairstyles
- Non-standard protective gear, such as uniquely designed masks

These challenges are common in vision-based systems and provide opportunities to enhance detection models through more diverse data and adaptive preprocessing techniques.

### 6.2 Security considerations

The current QR code-based vaccination verification framework provides a functional baseline but can benefit from additional security layers. For instance:

- Incorporating secure QR code generation with cryptographic validation (e.g., digital signatures) can help mitigate risks such as duplication or reuse of codes.
- Enhancing the existing Firebase infrastructure with features like token expiration, backend payload verification, and two-factor authentication for administrative access can further strengthen system integrity.

These enhancements would align the system with best practices in secure health data handling.

### 6.3 Deployment constraints

The system has been evaluated in a controlled setting with stable lighting and connectivity. To support broader adoption, it is important to evaluate performance under:

- Dynamic or high-traffic environments (e.g., public events or institutional entrances)
- Outdoor conditions with variable lighting and motion
- Network-limited scenarios where offline operation or edge processing may be required

While current results are encouraging, deployment at scale may require hardware optimization and backend scaling to maintain responsiveness.

Future Work

Future enhancements of the system can explore the following directions:

- Real-world deployment trials involving diverse participant groups and uncontrolled settings
- Use of secure, tamper-resistant QR codes (e.g., tokenized or blockchain-backed solutions)
- Integration of liveness detection techniques (e.g., eye-blink or motion-based verification) to prevent spoofing
- Expansion of the training dataset to include edge cases, such as partially worn masks, various age groups, and diverse ethnic backgrounds
- Performance benchmarking across different edge hardware configurations to guide optimization for resource-constrained environments

Overall, the proposed system offers a viable and adaptable framework for real-time public health screening. With continued improvements in security, detection robustness, and deployment readiness, it holds significant potential for broader application in both institutional and public settings.

## 7 Conclusion

The proposed IoT- and machine learning-based framework addresses the challenges of infectious disease management in communal spaces by integrating multiple subsystems, including face mask and face shield detection, contactless sanitization, temperature monitoring, vaccination certificate verification, and real-time data processing. Leveraging a hybrid model combining MobileNetV2 and VGG19, the system achieves high detection accuracies for face masks and face shields, while maintaining computational efficiency and suitability for real-time deployment. The integration of QR-based vaccination verification, a contactless temperature monitoring system, and a Google Firebase real-time database ensures seamless entry management with minimal latency. Comparative analysis with existing systems demonstrates superior performance in accuracy, processing speed, and feature integration, establishing its efficacy as a scalable and robust public health solution. The modular design allows adaptability to various health crises, making it a valuable tool for mitigating the spread of infectious diseases. Future research can focus on extending the dataset, integrating additional functionalities, and optimizing system performance to cater to evolving public health needs. This work highlights the potential of AI and IoT in enhancing public safety and establishes a foundation for developing intelligent systems for communal health monitoring and infectious disease prevention.

## Data Availability

The original contributions presented in the study are included in the article/supplementary material, further inquiries can be directed to the corresponding authors.

## References

[1] Cui C Li B Chen X. Group decision-making method of entry policy during a pandemic. Tsinghua Sci Technol. (2024) 29:56–65. 10.26599/TST.2022.9010014

[2] Shukla SSP Jain VK Yadav AK Pandey SK. Analyzing COVID-19 using mathematical SEIRS epidemic models and deep neural networks. Multimedia Tools and Applications. (2023). 10.1007/s11042-023-16609-x

[3] Khan JI Khan J Ali F Ullah F Bacha J Lee S. Artificial intelligence and internet of things (AI-IoT) technologies in response to the COVID-19 pandemic: a systematic review. IEEE Access. (2022) 10:62613–60. 10.1109/ACCESS.2022.3181605

[4] Kumar KR Meenakshisundaram I Niveditha VR Magesh S Magesh G Marappan S.. Monitoring and analysis of the recovery rate of COVID-19 positive cases using IoT and sensors. Int. J. Pervasive Comput. Commun. (2022) 18, 365–375. 10.1108/IJPCC-07-2020-0088

[5] Iyer R Ringe PS Iyer VR Bhensdadiya KP. Comparison of YOLOv3, YOLOv5s, and MobileNet-SSD V2 for real-time mask detection. Int. J. Res. Eng. Technol. (2023) 8:1156–60.

[6] Rajasekhar AHN Kavya G Sriya K Belwal M. Face mask detection using CNN via active learning. IEEE Intelligent Technologies Conference Proceedings. (2023).

[7] Gautam M Chaudhary H Verma A Garg R. Automated COVID-19 detection using ML and IoT. IoT Commun Autom Technol J. (2023) 6:112–123. 10.1109/ICICAT57735.2023.10263743

[8] Mbunge E Simelane S Fashoto SG Akinnuwesi B Metfula AS. Application of deep learning and machine learning models for COVID-19 face mask detection. Sustain Oper Comput. (2023) 2:235–45. 10.1016/j.susoc.2021.08.001

[9] Pertsau D Uvarov A. Face detection algorithms in hybrid IoT systems. Comput Vis Image Underst. (2023) 117:45–55.

[10] Stolojescu-Crisan C Crisan C Butunoi BP. An IoT-based smart home automation system. Sensors. (2022) 21:56–67. 10.3390/s21113784 34070717 PMC8198920

[11] Shashank Reddy S. Temperature monitoring system using IoT and telegram alerts. Int J Interdiscip Innov Res Dev. (2022) 5:156–8.40292876

[12] Whitfield CA Van Tongeren M Han Y Wei H Daniels S Regan M. Modeling the impact of non-pharmaceutical interventions on workplace transmission of SARS-CoV-2 in the home-delivery sector. PLoS One. (2023) 18:e0284805. 10.1371/journal.pone.0284805 37146037 PMC10162531

[13] Rajasekhar AHN Kavya G Sriya K Belwal M. Face mask detection using CNN via active learning, 2023 3rd International Conference on Intelligent Technologies (CONIT), Hubli, India (2023) 1–7. 10.1109/CONIT59222.2023.10205914

[14] Shashank Reddy S. Temperature monitoring and alert system using BoltIoT and telegram. Int J Interdiscip Innov Res Dev (IJIIRD) (2020) 5.40292876

[15] Dutta P Dutta P. A cheaper home automation using BoltIoT. Int J Sci Res Dev (IJSRD). (2019) 7:1433–5.

[16] Naufal MF Kusuma SF Prayuska ZA Yoshua AA Lauwoto YA Dinata NS. Comparative analysis of image classification algorithms for face mask detection. J Inform Sys Eng Bus Intel. (2021) 7:56–66. 10.20473/jisebi.7.1.56-66

[17] Iyer R Shashikant Ringe P Varadharajan Iyer R. Comparison of YOLOv3, YOLOv5s and MobileNet-SSD V2 for real-time mask detection. Artic Int J Res Eng Technol. (2021) 8:1156–60.

[18] Dilu KP Navya Martin Sreelakshmi KS. IoT based contactless body temperature measurement and data collection for COVID-19. 2nd International Conference on IoT Based Control Networks and Intelligent Systems (ICICNIS) (2021).

[19] Tu CH Lee JH Chan YM Chen CS. Pruning depthwise separable convolutions for mobilenet compression. In 2020 International Joint Conference on Neural Networks (IJCNN) IEEE (2020). pp. 1–8. 10.1109/IJCNN48605.2020.9207259

[20] Sinha D El-Sharkawy M. Thin mobilenet: an enhanced mobilenet architecture. In 2019 IEEE 10th annual ubiquitous computing, electronics and mobile communication conference (UEMCON) IEEE (2019) pp. 0280–0285. 10.1109/UEMCON47517.2019.8993089

[21] Sae-Lim W Wettayaprasit W Aiyarak P. Convolutional neural networks using MobileNet for skin lesion classification. In 2019 16th international joint conference on computer science and software engineering (JCSSE) IEEE (2019). pp. 242–247. 10.1109/JCSSE.2019.8864155

[22] Suresh K Palangappa M Bhuvan S. Face Mask Detection by using Optimistic Convolutional Neural Network, 2021 6th International Conference on Inventive Computation Technologies (ICICT). (2021) 1084–1089. 10.1109/ICICT50816.2021.9358653

[23] Balaji S Balamurugan B Kumar TA Rajmohan R Kumar PP. A brief survey on AI based face mask detection system for public places. Irish Interdisciplinary Journal of Science and Research (IIJSR). (2021).

[24] Pooja S. Preeti S. Face mask detection using AI. In Predictive and Preventive Measures for Covid-19 Pandemic. Singapore: Springer (2021). pp. 293-305 10.1007/978-981-33-4236-1_16

[25] Nowrin A Afroz S Rahman MS Mahmud I Cho YZ. Comprehensive review on facemask detection techniques in the context of covid-19. IEEE access. (2021). 10.1109/ACCESS.2021.3100070

[26] Sanjaya SA Rakhmawan SA. Face mask detection using MobileNetV2 in the era of COVID-19 pandemic. In 2020 International Conference on Data Analytics for Business and Industry: Way Towards a Sustainable Economy (ICDABI) IEEE. (2020). pp. 1–5. 10.1109/ICDABI51230.2020.9325631

[27] Jiang X Gao T Zhu Z Zhao Y. Real-time face mask detection method based on YOLOv3. Electronics. (2021) 10:837. 10.3390/electronics10070837

[28] Wu P Li H Zeng N Li F. FMD-Yolo: an efficient face mask detection method for COVID-19 prevention and control in public. Image Vis Comput. (2022) 117:104341. 10.1016/j.imavis.2021.104341 34848910 PMC8612756

[29] Susanto S Putra FA Analia R Suciningtyas IKLN. The face mask detection for preventing the spread of COVID-19 at Politeknik Negeri Batam. In 2020 3rd International Conference on Applied Engineering (ICAE) IEEE. (2020). pp. 1–5. 10.1109/ICAE50557.2020.9350556

[30] Kong J Deng Y. GPU accelerated face detection. In 2010 International Conference on Intelligent Control and Information Processing IEEE (2010). pp. 584–588. 10.1109/ICICIP.2010.5564978

[31] Reza SR Dong X Qian L. Robust Face Mask Detection using Deep Learning on IoT Devices. In 2021 IEEE International Conference on Communications Workshops (ICC Workshops) IEEE (2021). pp. 1–6. 10.1109/ICCWorkshops50388.2021.9473701

[32] Martínez-Zarzuela M Díaz-Pernas FJ Antón-Rodríguez M Perozo-Rondón F González-Ortega D. AdaBoost face detection on the GPU using Haar-like features. In International Work-Conference on the Interplay Between Natural and Artificial Computation. Berlin, Heidelberg: Springer (2011). pp. 333–342. 10.1007/978-3-642-21326-7_36

[33] Pertsau D Uvarov A. Face detection algorithm using haar-like feature for GPU architecture. In 2013 IEEE 7th International Conference on Intelligent Data Acquisition and Advanced Computing Systems (IDAACS) IEEE (2013). Vol. 2, pp. 726–730. 10.1109/IDAACS.2013.6663020

[34] Viola P Jones M. Rapid Object Detection using a Boosted Cascade of Simple Features. IEEE Conference on Computer Vision and Pattern Recognition (CVPR). (2001) 990517. 10.1109/CVPR.2001.990517

[35] Nolan D Lang DT. “Javascript object notation,” In: XML and Web Technologies for Data Sciences with R. (New York, NY: Springer) (2014). pp. 227–253. 10.1007/978-1-4614-7900-0_7

[36] Habib S Alsanea M Aloraini M Al-Rawashdeh HS Islam M Khan S. An efficient and effective deep learning-based model for real-time face mask detection. Sensors. (2022) 22:2602. 10.3390/s22072602 35408217 PMC9003465

[37] Goyal H Sidana K Singh C Jain A Jindal S. A real time face mask detection system using convolutional neural network. Multimed Tools Appl. (2022) 81:14999–5015. 10.1007/s11042-022-12166-x 35233179 PMC8874748

